# Spatial proteomics in translational and clinical research

**DOI:** 10.1038/s44320-025-00101-9

**Published:** 2025-04-14

**Authors:** Peter Horvath, Fabian Coscia

**Affiliations:** 1 Synthetic and Systems Biology Unit, Biological Research Centre, HUN-REN, Szeged, Hungary; 2 Single-Cell Technologies Ltd., Szeged, Hungary; 3 Institute of AI for Health, Helmholtz Zentrum München, Neuherberg, Germany; 4 Max-Delbrück-Center for Molecular Medicine in the Helmholtz Association (MDC), Spatial Proteomics Group, Berlin, Germany

**Keywords:** Proteomics

## Abstract

This Comment discusses recent advances in spatial proteomics, explores emerging multi-scale approaches for tissue profiling, and highlights their translational potential.

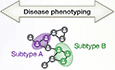

## Teaser

Spatial proteomics offers unprecedented insights into the localization, quantity, and interactions of proteins within cells and tissues, thereby enabling researchers and clinicians to map protein networks with high precision. This powerful approach is currently revolutionizing our understanding of cellular organization and function (Method of the Year 2024: spatial proteomics, 2024), and providing deeper insights into how protein distribution and dynamics contribute to physiological and pathological processes. Here, we discuss recent advances in targeted and untargeted (exploratory) spatial proteomics, shed light on emerging multiscale approaches for tissue profiling, and highlight their translational potential.

## Technology snapshot

Spatial proteomics (SP) encompasses a diverse array of technologies that vary in scale, resolution, and sensitivity, offering researchers and clinicians a broad range of tools to investigate the spatially resolved proteome. SP has emerged to bridge the gap between genomic insights and the direct study of proteins within their native tissue environments. Although single-cell and spatial transcriptomics have offered significant insights into cellular diversity, these techniques do not effectively capture the dynamic behavior of proteins, which are the main controllers of biological processes.

SP methods can be broadly categorized into targeted and untargeted (exploratory) approaches.

While targeted SP are generally antibody-based, exploratory methods rely on mass spectrometry (MS) and can be further divided into MALDI mass spectrometry imaging (MSI) and liquid chromatography (LC)-MS.

### Antibody-based spatial proteomics

Antibody-based approaches have been widely used to detect protein distribution via chromogenic and fluorescence signals. Conventional methods, such as immunohistochemistry (IHC) and immunofluorescence (IF), have evolved into highly multiplexed imaging technologies, allowing the simultaneous mapping of dozens of proteins within tissue sections. Advanced techniques such as cyclic immunofluorescence (CycIF), co-detection by indexing (CODEX), and Imaging Mass Cytometry (IMC) now enable the spatial localization of more than 50 proteins at subcellular resolution. However, limitations such as spectral overlap, tissue autofluorescence, and long staining-imaging cycles hinder scalability.

Recent developments have introduced DNA-barcoded antibodies, which improve detection capabilities by enabling single-molecule resolution. Technologies such as Spatial CITE-seq allow for the simultaneous mapping of RNA and protein expression, significantly enhancing multiomics integration. Metal-labeled antibodies, used in methods such as multiplexed ion beam imaging (MIBI-TOF) and IMC, provide high-throughput spatial proteomic mapping with superior sensitivity.

### Mass spectrometry imaging (MSI) for spatial proteomics

Exploratory MSI provides an antibody-free alternative for spatial proteomics, generating protein maps directly from tissue sections. There are two major strategies to achieve this goal.

Top-down proteomics: Intact proteins are ionized and analyzed directly, allowing for the study of proteoforms and post-translational modifications (PTMs). MALDI-IMS has been used to map histone modifications and high-molecular-weight proteins.

Bottom-up proteomics: This method involves enzymatic digestion of proteins into peptides, enhancing sequence coverage. Advances, such as in situ tryptic digestion and integration with high-resolution mass spectrometry, have improved the sensitivity and applicability of clinical formalin-fixed paraffin-embedded (FFPE) samples.

### LC-MS-based spatial proteomics

LC-MS-based proteomics has historically been the key tool for unbiased protein identification, and recent technological advances have enabled its application in spatial and single-cell proteomics (Mund et al, 2022a). Two main strategies have been developed.

Grid-based analysis: This method involves dividing the tissue into small voxels, followed by protein extraction and LC-MS analysis. Early studies in 2007 demonstrated spatial proteomics in brain tissue, detecting over 1000 proteins per voxel (Petyuk et al, 2007). Recent innovations, such as nanoPOTS and 3D-printed microscaffolds, have improved sensitivity, enabling the detection of a few thousand proteins at 50–100 µm resolution.

Region of interest (ROI) selection: In contrast to grid-based approaches, ROI-based techniques rely on laser microdissection (LMD) to isolate specific areas of interest (e.g., single cells or cellular niches), thereby reducing sample preparation complexity while maintaining spatial resolution.

## Towards multiscale and multimodal

The integration of targeted and exploratory spatial proteomics holds immense promise for basic and translational research. Their synergy lies in their complementary strengths. Targeted methods excel in focused analyses of known proteins, typically up to 50 markers, at high cell throughput and (sub)cellular resolution, whereas exploratory MS-based techniques enable novel discoveries without prior information. However, exploratory methods are still limited in terms of cell throughput, and are ideally applied using screening-type targeted approaches. This combination (referred to as the multiscale approach) enables a more comprehensive understanding of the spatial organization and functional state of diverse cell types in complex tissues. Multiscale approaches are still in their infancy and have only recently become achievable through advances in computational image analysis coupled with single-cell isolation in situ (Brasko et al, 2018), as well as ultrasensitive MS-based proteomics (Bennett et al, 2023). A prominent example is deep visual proteomics (DVP) (Mund et al, 2022b), which harnesses the synergy among high-resolution microscopy, AI-guided image analysis, and laser microdissection (LMD)-empowered deep proteomic profiling. This framework allows researchers to visualize, quantify, and correlate protein levels, subcellular localization, and post-translational modifications within a single archival tissue section. Its potential in precision medicine was demonstrated in a fatal skin disease termed toxic epidermal necrolysis (TEN) (Nordmann et al, 2024). The research team identified a critical role for the JAK/STAT pathway in TEN and uncovered JAK/STAT inhibition as a promising curative treatment strategy, with demonstrated recovery in seven patients. Other multiscale pipelines include SCPro, FAXP, MALDI-MS imaging combined with antibody-coupled mass reporters, LMD and LC-MS-based proteomics. With rapid advances in computational image analysis, automated sample processing pipelines, and ultrasensitive MS, it can be expected that other innovative and powerful multiscale concepts are already being developed.

The development of multiscale pipelines provides an exciting entry point for spatial multimodal concepts by combining proteomics with other omics techniques (genomics, transcriptomics, epigenomics, and metabolomics). This is because multiomics strategies rely equally on machine learning approaches capable of handling and integrating different data types and formats. Multiomics data integration can reveal the impact of genetic, epigenetic, and transcriptional variations on protein abundance, providing a more holistic view of disease-causing processes and active biological pathways. Although multimodal data from the same tissue slide are generally preferable, such concepts are currently only possible for targeted SP. However, serial tissue sections offer a reasonable alternative, as specific technological requirements are met without the development of new sample-processing protocols. Different data layers can be computationally combined and overlaid, for example, by using H&E staining and recurring tissue anchor points. Several open-source software pipelines and data formats have already been developed to facilitate seamless data integration (e.g., SpatialData) (Marconato et al, 2024). In addition, innovative new discovery concepts integrate spatial transcriptomics and deep MS-based proteomics through transfer learning (Hu et al, 2025). Such concepts are also promising for the integration of targeted and untargeted SP data, as they offer the ability to infer quantitative protein information of individual cell types and states without the need to acquire extensive exploratory data (i.e., thousands of MS measurements).

## New tools—new questions

The development of multiscale SP pipelines has raised interesting new questions regarding experimental design. For example, what is the ideal target cell number and proteomic coverage required to uncover critical disease mechanisms? This decision involves balancing the deep proteome coverage and spatial resolution with practical limitations, such as phenotype abundance, cohort size, and available LC-MS infrastructure. Optimized pipelines currently quantify 4000–6000 proteins from only 50 phenotype-matched cells, already providing sufficient proteome coverage to assess functional cell states driving health and disease. Assuming a similar pace of current technological advances, proteome-wide spatial analyses (i.e., 10,000 unique proteins or more) of small cellular niches could become a reality within a few years.

Furthermore, does integrating cell type-resolved deep proteomic data justify the design of smaller targeted antibody panels in the future? This could save time and resources and lower computational demands. Panels of 20–30 proteins could offer a good compromise for spatial phenotyping of major cell types and their interactions, while deep proteomes of selected cell types would illuminate their functional state. However, how can we best select the cell types and states to focus on for deep exploratory analysis? Machine learning algorithms trained on imaging, other omics and clinical data can identify phenotypes statistically associated with clinical metadata, such as therapeutic responses and survival outcomes (de Souza et al, 2024). Exploratory SP then enable ‘zooming in’ on critical cell populations and provide unprecedented insights into the quantitative and functional state of the proteome, offering a treasure trove for biomarker and drug target discovery. Alternatively, prior knowledge of disease-driving cell types can guide target cell selection in a more supervised fashion.

## Clinical impact and future directions

**Figure 1:**
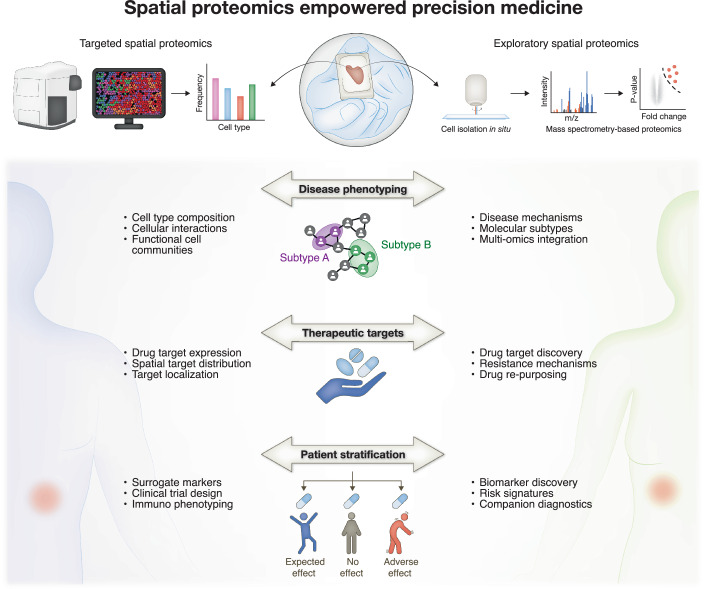
Spatial proteomics empowered precision medicine. Targeted and exploratory spatial proteomics methods provide highly complementary data and promise to revolutionize precision medicine. Key applications include disease phenotyping, therapeutic targets, and patient stratification.

The clinical impact of spatial tissue proteomics is growing rapidly, with applications ranging from cancer biology to neurodegenerative disorders and infectious diseases. We expect three main application areas in precision medicine where targeted and exploratory SP will have a transformative impact over the next 5–10 years, particularly when applied in a concerted fashion (Fig. 1).

### Disease phenotyping

Traditional bulk proteomics approaches are powerful tools for mapping the global protein landscape of diseases and are crucial for identifying recurring molecular subtypes, prognostic and predictive risk signatures, and novel therapeutic targets. However, bulk-based phenotyping fails to capture the spatial heterogeneity of protein distribution within tissues, which adds a critical dimension to our understanding of disease pathogenesis. Exploratory SP data provide a more finely resolved picture and incorporate critical information regarding the tissue architecture, cellular composition, and spatial location of disease-driving phenotypes. Optimized workflows tailored to ultra-low sample amounts offer an exciting path for next-level tissue phenotyping, as they will soon provide comparable proteome coverage to bulk tissue workflows, which typically require hundreds of thousands of cells. The ability to profile a few cells makes this approach well-suited to study early disease states, such as precancerous lesions, and to identify their proteomic deviations from normal cells. This approach is key to developing preventative medicine strategies and identifying effective therapies targeting proteins expressed by most subclones. A promising future direction for SP-empowered disease phenotyping has been elucidated through targeted SP data, which has identified recurring, functionally diverse cell communities across disease entities. These recurring cellular modules may play similar roles in multiple diseases and offer potential targets for therapeutic interventions. However, our understanding of such functional niches remains limited and presents an exciting opportunity for exploratory proteomics to uncover their global, functional, and potentially actionable states.

### Therapeutic targets

The field of drug target discovery relies heavily on proteins, which constitute the majority of drug targets. Therapeutic agents are designed to interact with specific proteins expressed by cells within diseased tissues to modulate their function, alleviate symptoms, or treat underlying conditions. Hence, spatial proteomics offers a valuable methodology for gaining detailed insights into the precise localization and distribution of protein-based drug targets within cells and tissues. Targeted spatial proteomics has been extensively applied in the immuno-oncology field to characterize responder and non-responder patients receiving immune checkpoint inhibitors (ICI). It has become clear that single marker analyses (e.g., PD-L1 levels) are insufficient to accurately predict therapeutic outcomes, and that the composition and state of the tumor microenvironment needs more attention in clinical diagnostics. Despite growing knowledge of the cell types, interactions, and functional states associated with beneficial ICI responses, a clear mechanistic understanding of the underlying therapeutic outcomes is still lacking for most tumors. The combination with exploratory SP could offer a promising strategy to shed light on the active biological pathways driving complex resistance programs and illuminate alternative therapeutic targets known in different disease contexts. Such an approach might be best realized in large retrospective and prospective cohorts and by paired proteomic analyses comparing healthy cells to diseased cells within the same tissue sample.

### Patient stratification

The ability to profile spatial protein distributions in patient-derived samples has enabled the identification of prognostic and predictive biomarkers to guide personalized treatment strategies. Risk signatures can be developed by integrating spatial protein expression patterns with clinical outcomes, enabling more accurate patient risk assessment. For companion diagnostics, deep spatial proteomic analyses can reveal protein markers indicative of treatment response and guide therapy selection. A fast-track option to translate such findings into clinical practice could involve the integration of new surrogate IHC markers or small IF panels into routine histopathology. This approach leverages the existing infrastructure while capitalizing on the latest biological insights from exploratory data. In the long term, mass spectrometry-based assays using heavy spike-in standards can also be realized. For example, targeted MS panels can be designed to systematically profile all actionable proteins, including kinases, PTMs, and key oncogenic drivers in a tissue sample. Finally, spatial proteomics has great potential for guiding clinical trial design by identifying patient subgroups that are most likely to benefit from specific treatments. This approach enables targeted patient selection, potentially improves trial outcomes, and accelerates drug development.

### Future directions

The field of spatial proteomics is rapidly advancing, with efforts focused on improving its sensitivity, automation, and integration with other omics technologies. The development of single-cell proteomics atlases and spatial PTM profiling will further enhance our understanding of cellular architecture and disease mechanisms. AI-powered analytical tools play a crucial role in standardizing data processing and facilitating large-scale clinical application. Jointly, these advances will enable a deeper understanding of disease heterogeneity and treatment responses and promise to revolutionize personalized medicine.
